# Population genetic structure of the Asian bush mosquito, *Aedes japonicus* (Diptera, Culicidae), in Belgium suggests multiple introductions

**DOI:** 10.1186/s13071-021-04676-8

**Published:** 2021-03-25

**Authors:** Nathalie Smitz, Katrien De Wolf, Isra Deblauwe, Helge Kampen, Francis Schaffner, Jacobus De Witte, Anna Schneider, Ingrid Verlé, Adwine Vanslembrouck, Wouter Dekoninck, Kenny Meganck, Sophie Gombeer, Ann Vanderheyden, Marc De Meyer, Thierry Backeljau, Doreen Werner, Ruth Müller, Wim Van Bortel

**Affiliations:** 1 Royal Museum for Central Africa (BopCo & Biology Department), Leuvensesteenweg 17, 3080 Tervuren, Belgium; 2 The Unit of Entomology, Department of Biomedical Sciences, Institute of Tropical Medicine, Nationalestraat 155, 2000 Antwerp, Belgium; 3 Friedrich Loeffler Institut, Federal Research Institute for Animal Health, Südufer 10, 17493 Greifswald-Insel Riems, Germany; 4 Francis Schaffner Consultancy, Riehen, Switzerland; 5 Royal Belgian Institute of Natural Sciences (BopCo & Scientific Heritage Service), Vautierstraat 29, 1000 Brussels, Belgium; 6 Evolutionary Ecology Group, University of Antwerp, Universiteitsplein 1, 2610 Antwerp, Belgium; 7 Leibniz Centre for Agricultural Landscape Research, Eberswalder Straße 84, 15374 Müncheberg, Germany; 8 Outbreak Research Team, Institute of Tropical Medicine, Nationalestraat 155, 2000 Antwerp, Belgium

**Keywords:** *Aedes japonicus japonicus*, Introduction, Invasive mosquito, Population genetics, Temporal changes, Microsatellites, *Nad*4 haplotypes

## Abstract

**Background:**

*Aedes japonicus japonicus* has expanded beyond its native range and has established in multiple European countries, including Belgium. In addition to the population located at Natoye, Belgium, locally established since 2002, specimens were recently collected along the Belgian border. The first objective of this study was therefore to investigate the origin of these new introductions, which were assumed to be related to the expansion of the nearby population in western Germany. Also, an intensive elimination campaign was undertaken at Natoye between 2012 and 2015, after which the species was declared to be eradicated. This species was re-detected in 2017, and thus the second objective was to investigate if these specimens resulted from a new introduction event and/or from a few undetected specimens that escaped the elimination campaign.

**Methods:**

Population genetic variation at *nad*4 and seven microsatellite loci was surveyed in 224 and 68 specimens collected in Belgium and Germany, respectively. German samples were included as reference to investigate putative introduction source(s). At Natoye, 52 and 135 specimens were collected before and after the elimination campaign, respectively, to investigate temporal changes in the genetic composition and diversity.

**Results:**

At Natoye, the genotypic microsatellite make-up showed a clear difference before and after the elimination campaign. Also, the population after 2017 displayed an increased allelic richness and number of private alleles, indicative of new introduction(s). However, the Natoye population present before the elimination programme is believed to have survived at low density. At the Belgian border, clustering results suggest a relation with the western German population. Whether the introduction(s) occur via passive human-mediated ground transport or, alternatively, by natural spread cannot be determined yet from the dataset.

**Conclusion:**

Further introductions within Belgium are expected to occur in the near future, especially along the eastern Belgian border, which is at the front of the invasion of *Ae. japonicus* towards the west. Our results also point to the complexity of controlling invasive species, since 4 years of intense control measures were found to be not completely successful at eliminating this exotic at Natoye.
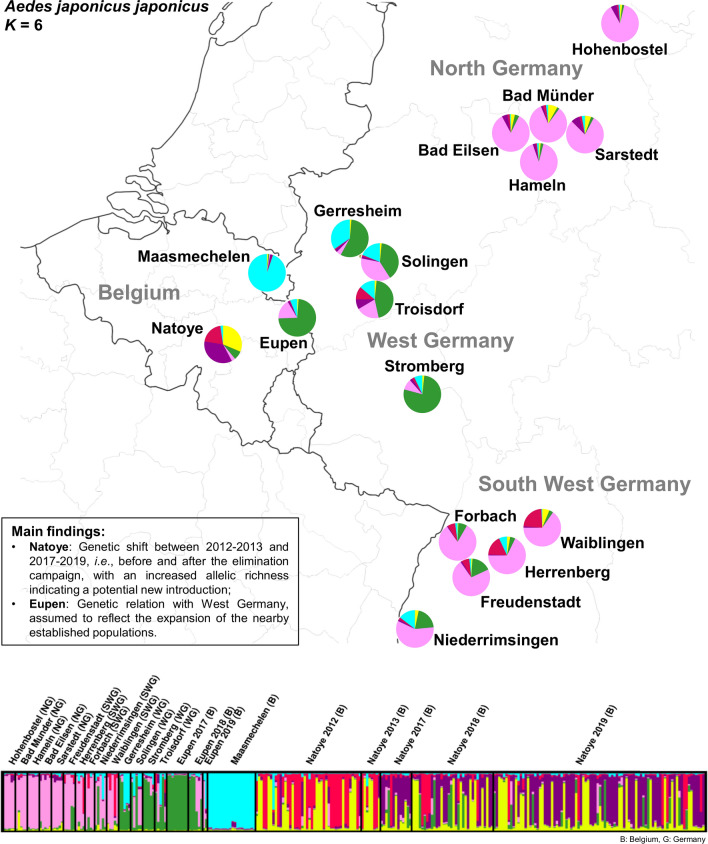

**Supplementary Information:**

The online version contains supplementary material available at 10.1186/s13071-021-04676-8.

## Background

As a result of globalisation and international trade, non-native species are being introduced into Europe, which may eventually establish reproducing and overwintering populations in new territories. The introduction of potential disease vectors is of major concern since these constitute a threat to human and animal health. Mosquitoes (Diptera: Culicidae), such as *Aedes* species, are regularly introduced together with the worldwide transport of used tyres, ornamental plants and water-holding machinery [1]. The Asian bush mosquito (*Aedes japonicus japonicus* (Theobald, 1901), generic name following [2]), is a competent vector in the laboratory for a number of arboviruses [3], including the West Nile [4], Japanese encephalitis [5], chikungunya [6], dengue [6] and Zika viruses [7, 8]. Originally restricted to East Asia, the species is well adapted to the temperate climates of Europe where it is now well-established [9, 10]. The arrival and spread of this species in central and western Europe has been attributed to its broad ecological tolerance, adaptability, low grade of specialisation in the choice of breeding sites and to its eggs withstanding desiccation and low temperatures [11–14]. The expansion and colonisation of new territories by the species is primarily passive and associated with human activities [1, 15].

Since the first detection of *Ae. japonicus* in Belgium in 2002 (at Natoye, municipality Hamois, Namur province) [11], successive monitoring projects have surveyed the introduction and spread of this and other exotic mosquito species [16–18]. Natoye was the first place in western Europe where the species was found to be established [11]. Subsequently it was found in Switzerland, Germany and France in 2008 [19–21], in Slovenia and Austria in 2011 [22], in Hungary in 2012 [23], in Croatia and the Netherlands in 2013 [24–26], in Italy and Lichtenstein in 2015 [27] and in Spain and Luxembourg in 2018 [28, 29]. The population at Natoye is the only one in Europe with a well-documented introduction pathway. *Aedes japonicus* was most likely introduced through the second-hand tyre trade located at this site [11]. The exact origin, however, is unknown since imports arrived from various locations, including countries already colonised by the species, like the USA [30]. Its presence at Natoye was confirmed in 2003, 2004, 2007–2009 and 2012–2014, but the species was never caught outside a radius of 3.5 km around the premises of the tyre trading company [16–18, 30, 31]. Therefore, the population was considered to be established but not expanding. From 2012 to 2015, an intensive control campaign aimed at eliminating the species from Natoye (mainly mechanical source reduction and the use of larvicide), and since the species was not detected in 2015 and 2016, it was assumed to be eliminated [32]. However, in 2017–2019 *Ae. japonicus* re-appeared [Deblauwe et al., Monitoring of exotic mosquitoes in Belgium (MEMO): Final Report Phase 7 Part 1: MEMO results. Antwerp: NEHAP, unpublished report, 33, 34], raising the questions of whether new specimens had been introduced, and from where they originated, *i.e.,* whether they represented undetected survivors of the elimination and/or involved new colonisers from other source populations.

In contrast to the situation at Natoye, *Ae. japonicus* has rapidly spread throughout the southwest region of Germany, following its first observation in 2008 in the federal state of Baden-Wuerttemberg [35–37]. Its introduction pathway, however, is not clear. As the species has been monitored in Germany since 2010, its continuous spread and increasing population densities could be tracked [38, 39]. *Aedes japonicus* was subsequently detected in 2012 in the western region of the country (southern North Rhine-Westphalia and northern Rhineland Palatinate) [40], in 2013 in the northern part (southern Lower Saxony and northeastern North Rhine-Westphalia) [41] and finally in 2015 in the southeastern region (Upper Bavaria) [15]. It is now considered to be well-established and no longer eradicable [38]. The western German population has been spreading since 2012, and it was predicted that the species would cross the border with Belgium in the near future, possibly as early as 2016 [39]. Therefore, *Ae. japonicus* was monitored in Belgium between 2017 and 2019 at the Belgian–German parking lot of Lichtenbusch and along the road and highway between two cemeteries (Raeren and Rocherath) along the German border. Specimens were collected in an allotment garden in Eupen (province of Liège, Belgium) (Deblauwe et al., unpublished report). *Aedes japonicus* was also detected in 2018 during the monitoring of *Aedes koreicus*, another non-native mosquito species that has established in Belgium, in that same period at an industrial area in Maasmechelen (province of Limburg, Belgium) (Deblauwe et al., unpublished report). Hence, elucidating the relationships between these Belgian specimens and the western German population is of great interest to understand the introduction events in Belgium, and it might help customising surveillance and control efforts in Belgium.

To uncover the relationships between the geographically separated European populations of *Ae. japonicus*, several population genetic investigations have been conducted in the past [15, 24, 38, 42, 43]. Highly polymorphic DNA regions were used in these studies, such as those associated with microsatellites and the mitochondrial NADH dehydrogenase subunit 4 (*nad*4) locus. These DNA markers enabled researchers to study the population genetic structure of *Ae. japonicus* [15, 24, 38, 42] and the changes in allelic frequencies through space and time [43, 44], and revealed several independent long-distance introductions into Europe [42]. Only a few Belgian specimens (*N* = 18) collected at Natoye in 2008 and 2010 were included in these population genetic analyses, revealing that the Natoye population had the lowest genetic diversity of all populations examined [24, 42]. In Germany, the most recent study included specimens from the four above-mentioned geographically isolated populations (i.e. the southwestern, western, northern and southeastern populations), and identified two population clusters based on microsatellite data [43]. The specimens sampled in the west and southwest of Germany had high probabilities of belonging to each identified genotype group, respectively; those sampled in the north and southeast of Germany had mixed assignment probabilities. This latter study suggested that the western German cluster still had a uniform make-up, while admixture has occurred over time between the three other German populations, compared to previous results [15], with a human-mediated carry-over of individuals between regions [43].

The objectives of the present study were to determine: (i) if the mosquito specimens collected along the Belgian border were introduced from the nearby existing western German population, and (ii) if the population at Natoye resulted from a new introduction event and/or from a few undetected specimens that escaped elimination. To answer these questions, population genetic variation at *nad*4 and seven microsatellite loci was surveyed in two ways: (i) a comparison of allelic frequencies and haplotypic diversities between populations from Eupen, Maasmechelen, and reference material from Germany, to assess if the Eupen and Maasmechelen populations are linked to those from the western part of Germany, and (ii) a comparison of the genetic composition and diversity of the population at Natoye between 2012–2013 and 2017–2019, to assess potential effects of the elimination campaign.

## Methods

### Sampling

In total, 292 *Aedes j. japonicus* specimens from Belgium and Germany were incorporated in the present study (Table 1). Of these, 224 specimens were collected in the framework of successive projects undertaken to monitor the introduction and establishment of exotic mosquito species in Belgium [17, 18, 45]. Among these 224 specimens, a subset (*N* = 52) collected in 2012 and during a survey from 2013 to 2016 at Natoye was incorporated in this study to investigate the temporal fine-scale genetic structure changes at that location. During the latest monitoring project (Monitoring of Exotic Mosquito Species in Belgium [MEMO], 2017–2019), *Ae. japonicus* eggs, larvae and adults were collected at Natoye (location: used tyre-trade company, coordinates: 50°20′20.2″N, 5°02′43.7″E), Eupen (allotment garden) and Maasmechelen (industrial area) (Table 1) [Deblauwe et al., unpublished report, 33, 34]. Eggs collected in 2012 (*N* = 9) were reared in the laboratory to adults for morphological identification, while eggs collected in 2017 (*N* = 6) were identified by mitochondrial cytochrome oxidase I gene (*COI*) DNA barcoding [46], following [47] (GenBank accession numbers: MT418505-MT418508, MT418510, MT418511; 100% Barcode of Life Data System [BOLD] similarity percentages). Before species identification, eggs and larvae were transferred to absolute ethanol and stored at room temperature, while adults were stored dry at − 20 °C. Larvae and adults were morphologically identified following keys and species descriptions [48, 49].

**Table 1:** Sample information of the *Aedes japonicus japonicus* specimens, including their geographical origin and year of collection

Study investigation level	Country	State/Province	Location	Year	N_*nad*4_	N_M_	Life stage at collection	Surveillance project^a^
Eupen	Belgium	Liège	Eupen	2017	9	9	L: 9	MEMO (Deblauwe et al., unpublished report)
	Belgium	Liège	Eupen	2018	5	6	L: 2; A: 4	MEMO (Deblauwe et al., unpublished report)
	Belgium	Liège	Eupen	2019	2	2	L: 2	MEMO (Deblauwe et al., unpublished report)
Maasmechelen	Belgium	Limburg	Maasmechelen	2018	19	20	A: 20	MEMO (Deblauwe et al., unpublished report)
Natoye	Belgium	Namur	Natoye	2012	44	44	L: 35, E: 9	ExoSurv [17]
	Belgium	Namur	Natoye	2013	8	8	L: 8	Avia-GIS [18]
	Belgium	Namur	Natoye	2017	13	13	A: 4, L: 3, E: 6	MEMO (Deblauwe et al., unpublished report)
	Belgium	Namur	Natoye	2018	31	34	A: 20, L: 14	MEMO (Deblauwe et al., unpublished report)
	Belgium	Namur	Natoye	2019	80	88	A: 45, L: 43	MEMO (Deblauwe et al., unpublished report)
North	Germany	Lower Saxony	Hohenbostel	2017	5	5	L: 5	GMMP [51]
	Germany	Lower Saxony	Bad Münder	2017	5	5	L: 5	GMMP [51]
	Germany	Lower Saxony	Hameln	2017	5	5	L: 5	GMMP [51]
	Germany	Lower Saxony	Bad Eilsen	2017	5	5	L: 5	GMMP [51]
	Germany	Lower Saxony	Sarstedt	2017	5	5	L: 5	GMMP [51]
Southwest	Germany	Baden-Württemberg	Freudenstadt	2016	5	5	L: 5	GMMP [51]
	Germany	Baden-Württemberg	Herrenberg	2016	4	4	L: 4	GMMP [51]
	Germany	Baden-Württemberg	Forbach	2017	4	4	L: 4	GMMP [51]
	Germany	Baden-Württemberg	Niederrimsingen	2017	5	5	L: 5	GMMP [51]
	Germany	Baden-Württemberg	Waiblingen	2017	5	5	L: 5	GMMP [51]
West	Germany	North Rhine-Westphalia	Gerresheim	2016	5	5	L: 5	GMMP [51]
	Germany	North Rhine-Westphalia	Solingen	2016	5	5	L: 5	GMMP [51]
	Germany	Rhineland-Palatinate	Stromberg	2017	5	5	L: 5	GMMP [51]
	Germany	North Rhine-Westphalia	Troisdorf	2017	4	5	L: 5	GMMP [51]
Total					278	292		

N_*nad*4_, number of specimens for which NADH dehydrogenase subunit 4 (*nad*4) sequences were obtained; N_M_, number of individuals genotyped for the seven microsatellites investigated; A, adult; L, larvae; E, egg

^a^Surveillance project: GMMP, German Mosquito Monitoring Programme; MEMO, Monitoring of Exotic Mosquito Species in Belgium; ExoSurv, Implementation of surveillance of exotic mosquitoes in Belgium

Further, *Ae. j. japonicus* reference specimens (*N* = 68) from well-identified German population clusters based on microsatellite data [43] were included. These specimens were collected by visiting cemeteries in 2016 and 2017 (Table 1). They comprised larvae that were reared to adults in the laboratory and subsequently morphologically identified using a standard key [50]. Specimens from the southeastern German population were not available for the present study.

### DNA extraction and PCR amplification

DNA was extracted from legs, abdomens or eggs using either the NucleoSpin® Tissue DNA extraction kit (Macherey–Nagel, Düren, Germany) or the QIAamp DNA Micro kit (Qiagen, Hilden, Germany), following the manufacturers’ protocols, except that the elution volume was set to 70 µl.

A fragment of the *nad*4 locus was sequenced using published primers and PCR cycling conditions [52]. The PCR reaction was carried out in a final volume of 20 µl, with each reaction mixture containing 2 µl of DNA template, 2 µl of 10× buffer, 1.5 mM MgCl_2_, 0.2 mM dNTP, 0.4 µM of each primer and 0.03 U/µl of Platinum™ *Taq* DNA Polymerase (Invitrogen™ [Thermo Fisher Scientific], Carlsbad, CA, USA). PCR products and negative controls were run in a 1.5% agarose gel, using a UV transilluminator and the MidoriGreen™ Direct (NIPPON Genetics Europe GmbH, Düren, Germany) method. Positive PCR amplicons were subsequently purified using the ExoSAP-IT™ protocol, following the manufacturer’s instructions, and sequenced in both directions on an ABI 3230xl capillary DNA sequencer using BigDye Terminator v3.1 chemistry (Thermo Fisher Scientific, Waltham, MA, USA). The quality of the sequencing output was checked with Geneious® R11 (Biomatters Ltd., Aukland, New Zealand), following which strands were trimmed, corrected, translated into amino acids and assembled using the same software. Consensus sequences were extracted and aligned using ClustalW in Geneious® R11 (https://www.geneious.com).

Specimens were genotyped for seven microsatellite loci developed for *Ae. japonicus* [53], using the two multiplexes presented in [53], except for the OJ5F primer which was redesigned according to [44, 54]. The PCR reactions were carried out in a final volume of 10 μl, containing between 0.08 and 0.20 μl of each 10 μM diluted primer, 5 μl Multiplex *Taq* PCR Master Mix (Qiagen) and 2 μl of DNA. PCR conditions started with an initial activation step at 94 °C/15 min; followed denaturation (94 °C/30 s), annealing (54 °C/30 s) and extension (72 °C/30 s) for 30 cycles; and a final extension step at 60 °C for 30 min. PCR products were sized on a 3130XL Genetic Analyzer (Applied Biosystems, Foster City, CA, USA) using 2 μl of PCR product, 12 μl of Hi-Di™ formamide (Applied Biosystems) and 0.3 μl of GeneScan™ 500 LIZ size standard (Applied Biosystems). Length variation visualisation and determination were performed using Geneious® R11.

### *Nad*4 data analysis

Available *nad*4 sequences (*N* = 48) were downloaded from GenBank and then aligned with the *nad*4 consensus sequences generated in this study, as well as with one outgroup sequence of *Aedes aegypti*, using Geneious® R11. A rooted Neighbour-Joining (NJ) tree was constructed based on the HKY distance model implemented in Geneious® R11, with branch support assessed by 1000 bootstrap replicates.

We performed a pairwise comparison of nucleotide frequencies between populations using Wright’s *F*-statistics, as implemented in Arlequin v3.5 [55] (1000 random permutations for significance, with subsequent standard Bonferroni correction). The haplotype frequencies, the mean number of pairwise nucleotide differences (*k*) and average gene diversity over nucleotide positions (*H*) were calculated. A haplotype network was constructed using the minimum spanning network method (Minspnet in Arlequin v3.5), with default settings.

### Microsatellite data analysis

A multilocus Bayesian cluster analysis was performed using Structure v2.3.4, without prior information on geographic origin [56, 57]. A burn-in of 100,000 iterations and 1,000,000 Markov chain Monte Carlo method was applied. Each potential number of genotypic clusters (*K*; ranging from 1 to 10) was run ten times. The Markov chain convergence was checked between each ten iterations for each *K*. The results and visual output of the ten iterations for each *K* value were summarised using the web application CLUMPAK [58] (http://clumpak.tau.ac.il/index.html) and the software DISTRUCT v1.1 [59]. The optimal number of clusters was assessed following [60].

The presence of null alleles was tested with Micro-Checker v2.2.3 [61]. Heterozygosities (*H*_*e*_*, H*_*o*_) and inbreeding coefficient (*F*_*IS*_) per population were estimated using Genetix v4.05 [62], with 1000 permutations to calculate *P* values. The number of alleles (*N*), mean number of alleles per locus (*N*_*A*_) and number of private alleles (*P*_*A*_) per population were estimated using GenAlEx v6.51b2 [63]. Allelic richness (*A*_*R*_), as a standardised measure of the number of alleles per locus independent of the sample size, was calculated using FSTAT v2.9.4 [64]. Pairwise *F*_*ST*_ values between populations across all loci were estimated in Arlequin v3.5 (1000 permutations for significance, and subsequent standard Bonferroni correction). To further investigate the putative origin of the specimens collected along the Belgian border, a principal coordinates analysis (PCoA) was performed with GenAlEx v6.51b2 [63], based on Nei’s genetic distance and pairwise population *F*_*ST*_ values.

Recent demographic bottlenecks were explored with Bottleneck v1.2.02 [65] based on the Wilcoxon’s test under the stepwise mutation model to detect if loci showed a heterozygote excess or deficit. Significant heterozygote excesses may be indicative of a recent bottleneck [66].

## Results

The *nad*4 fragment was scored in 278 specimens (Table 1). The sequences were deposited in GenBank (accession numbers: MT462702—MT462979). The *nad*4 sequence alignment showed 15 transitions, all of which were silent. One new haplotype was discovered at Natoye (2019), and was named H47 (GenBank accession number: MT462840), in continuation of the numbering of *nad*4 haplotypes within the species [24, 43]. Heteroplasmy was identified based on the observation of double peaks in the sequence chromatograms. Because of these double peaks at specific nucleotide locations, as observed in previous studies within *Ae. japonicus* [24, 43]*,* 103 individuals could not be assigned to single haplotypes (Table 2). The amplification of nuclear insertions of mitochondrial origin (NUMTs) is considered to be unlikely because the detected polymorphic sites are located in the third codon position and are synonymous. Contaminations during laboratory procedures are also excluded since particular attention was given to avoid cross-contaminations, with repeated DNA extractions and PCR reactions performed under appropriate laboratory conditions. However, since sequencing is not the best way to reveal heteroplasmy in the mitochondrial genome, further investigations would be required.

**Table 2:** Number of specimens assigned to each *nad*4 haplotype at the different collection locations in Belgium and Germany

Collection locations^a^	*nad*4 haplotypes (*N*)^b^								mtDNA heteroplasmy (*N*)	Total *N*
	H1	H5	H6	H9	H10	H23	H46	H47		
Western Germany	10	5	1	0	0	0	0	0	3	19
Southwestern Germany	6	2	1	0	1	0	1	0	12	23
Northern Germany	0	0	0	2	9	0	0	0	14	25
Eupen 2017	9	0	0	0	0	0	0	0	0	9
Eupen 2018	0	1	4	0	0	0	0	0	0	5
Eupen 2019	2	0	0	0	0	0	0	0	0	2
Maasmechelen 2018	19	0	0	0	0	0	0	0	0	19
Natoye 2012	2	0	0	13	0	1	0	0	28	44
Natoye 2013	1	0	0	1	0	0	0	0	6	8
Natoye 2017	0	3	0	7	0	0	0	0	3	13
Natoye 2018	4	0	0	22	0	0	0	0	5	31
Natoye 2019	8	0	0	39	0	0	0	1	32	80

^a^Unless specified otherwise, locations are in Belgium

^b^Naming of haplotypes according to [24, 43]

Additionally, seven polymorphic microsatellite loci were scored in 292 specimens of *Ae. japonicus* (Table 1; 224 from Belgium and 68 from Germany). The number of alleles per locus and per population varied from 4 to 11, and from 15 to 37, respectively. The mean *H*_*e*_ ranged from 0.381 to 0.678, and the mean *H*_*o*_ from 0.384 to 0.609 (Table 3). Micro-Checker v2.2.3 did not detect null alleles. The microsatellite database is available from the Dryad Digital Repository ( https://doi.org/10.5061/dryad.p5hqbzkmw).

**Table 3:** Descriptive statistics of the genetic diversity within each population, and between sampling periods at Natoye, Belgium

Country	Region/locality	Microsatellites								*nad*4	
		*N*_*s*_	*N*	*N*_*a*_	*P*_*a*_	*A*_*R*_	*H*_*o*_ ± SD	*H*_*e*_ ± SD	*F*_*IS*_	*k* ± SD	*H* ± SD
Germany	Northern	25	29	4.143	3	4.093	0.480 ± 0.214	0.521 ± 0.196	0.081*	2.830 ± 1.539	0.007 ± 0.004
	Southwestern	23	37	5.286	4	4.983	0.609 ± 0.120	0.678 ± 0.093	0.105*	2.044 ± 1.186	0.005 ± 0.003
	Western	20	29	4.143	0	4.054	0.539 ± 0.125	0.593 ± 0.100	0.094*	0.813 ± 0.608	0.003 ± 0.003
Belgium	Eupen	17	24	3.429	0	3.429	0.538 ± 0.273	0.580 ± 0.118	0.075	0.925 ± 0.669	0.002 ± 0.002
	Maasmechelen	20	15	2.143	0	2.140	0.493 ± 0.380	0.381 ± 0.285	− 0.305*	0.000 ± 0.000	0.000 ± 0.000
	Natoye	187	33	4.714	2	3.800	0.384 ± 0.259	0.472 ± 0.238	0.186*	2.090 ± 1.173	0.006 ± 0.003
	Natoye (2012–2013)	52	23	3.286	1	3.286	0.421 ± 0.312	0.504 ± 0.292	0.167*	2.837 ± 1.518	0.007 ± 0.004
	Natoye (2017–2019)	135	32	4.571	10	4.292	0.370 ± 0.249	0.426 ± 0.215	0.132*	1.609 ± 0.960	0.004 ± 0.003

Statistics were calculated based on the database for the seven microsatellites, with Genetix v4.05 (*H*_*O*_*, H*_*e*_*, F*_*IS*_), FSTAT v2.9.4 (*A*_*R*_) and GenAlEx v6.51b2 (*N*_*a*_*, P*_*a*_)

*Significant difference, based on the *nad*4 database using Arlequin v3.5 (*k, H*)

*N*_*s*_, number of specimens; *N*, number of alleles; *N*_*a*_, mean number of alleles per locus; *P*_*a*_, number of private alleles; *H*_*o*_, observed heterozygosity; *H*_*e*_, unbiased expected heterozygosity; *F*_*IS*_, inbreeding coefficient; *k*, mean number of pairwise nucleotide differences; *H*, average gene diversity over nucleotide positions; *A*_*R*_, allelic richness with rarefaction to the common sample size of 17 and 52 individuals, estimated based on the whole genotype database, and between sampling periods at Natoye, respectively; SD, standard deviation

### Geographic analysis: introduction source

The NJ tree based on *nad*4 displayed an unresolved topology. Likewise, the minimum spanning network revealed no association between haplotypes and geography. The number of haplotypes per location varied from one (Eupen and Maasmechelen) to five (southwestern Germany). Haplotype H1 was encountered at almost all locations, and usually in higher frequencies (except at Natoye and in northern Germany), as elsewhere in the world [43, 52].

**Fig. 1:**
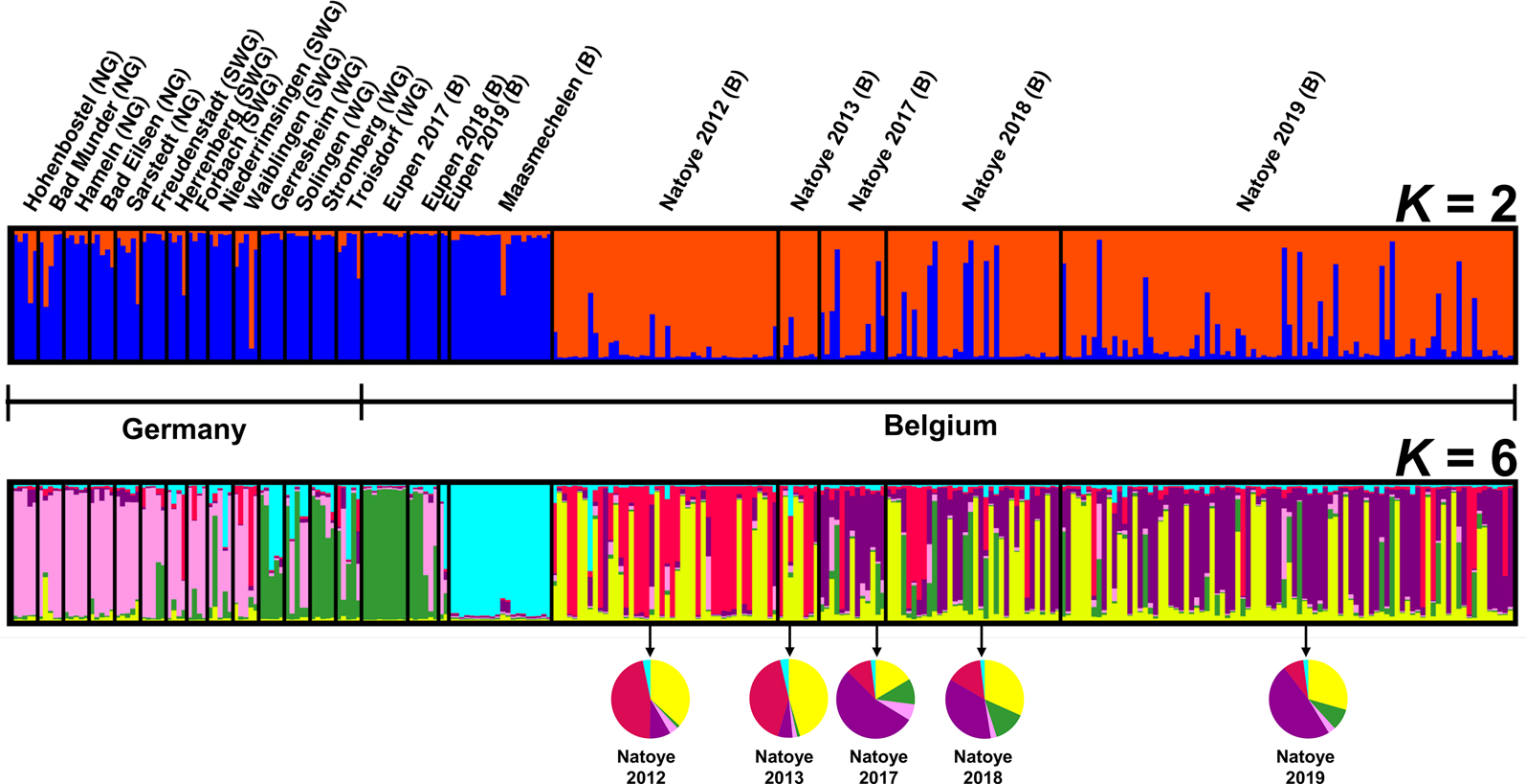
Clusters for both *K* = 2 and *K* = 6 (*K* = number of genotypic clusters), inferred with Structure v2.3.4 software, after Evanno et al*.* [60] correction. The cluster membership of each individual is shown by the colour composition of the vertical lines, with the length of each coloured part of the line being proportional to the estimated membership coefficient. WG, western Germany; SWG, southwestern Germany; NG, northern Germany; B, Belgium. Colours of the pie chart represent the mean assignment probabilities for all individuals collected at Natoye to each of the clusters per collection year

**Fig. 2:**
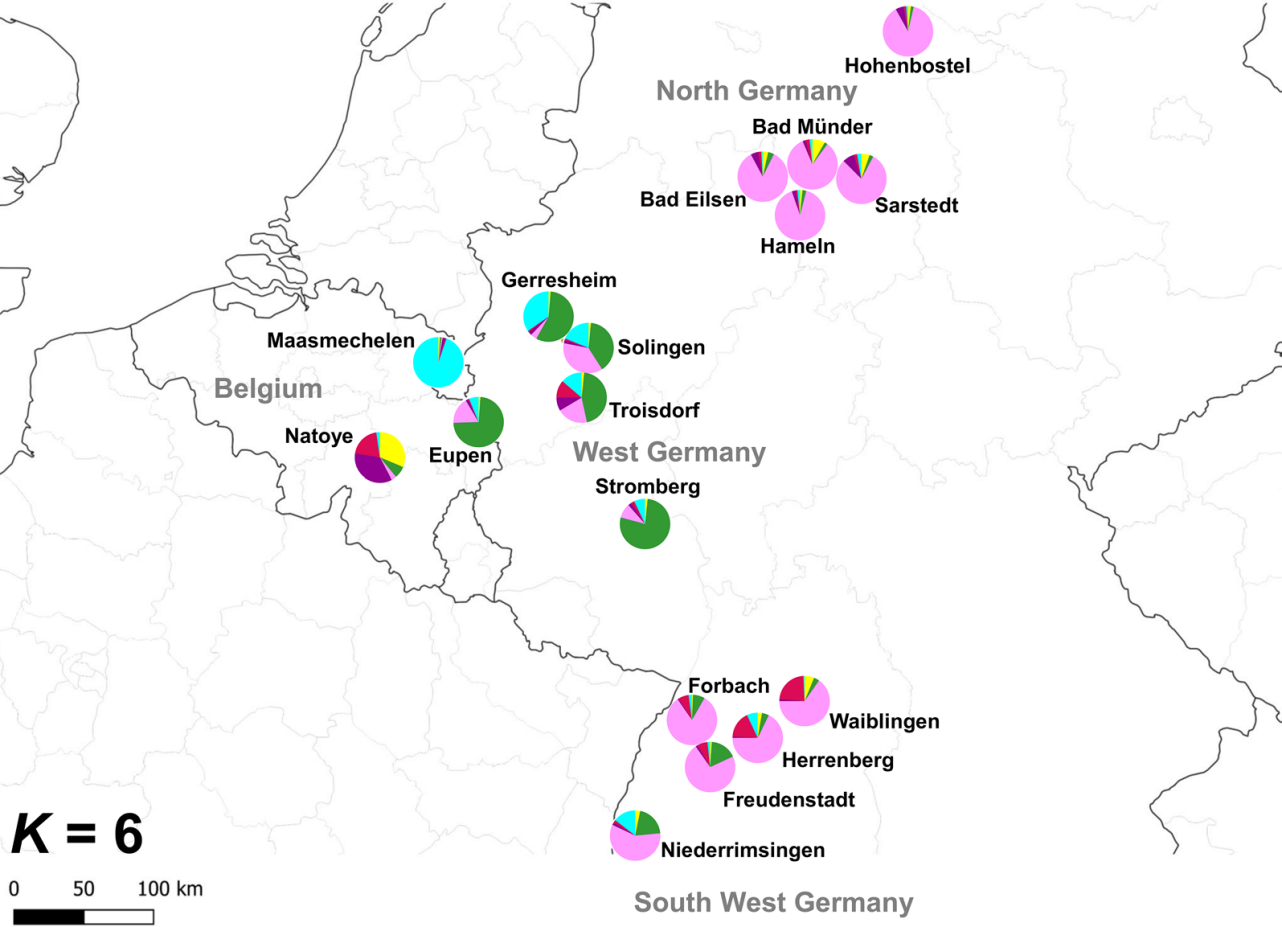
Map of Belgium and Germany showing the Bayesian cluster analysis results for *K* = 6 per sampling locality, based on seven microsatellite loci. Each pie chart represents one sampling location. Colours of the pie chart represent the mean assignment probabilities for all individuals collected at that location to each of the clusters, following the same colour code as in Fig. 1

Bayesian cluster analysis of the microsatellite data identified two (highest posterior probability for *K* = 2) and six (second highest posterior probability for *K* = 6) genotypic clusters (Fig. 1, Additional file 1: Fig. S1). At *K* = 2, the specimens from Natoye are separated from all others (Additional file 2: Fig. S2), and pairwise significant *nad*4 and microsatellite *F*_*ST*_ between Natoye and the other populations were 0.339 and 0.116 (*P* < 0.0005), respectively. At *K* = 6, four genotype groups corresponded with geographical populations, with different degrees of admixture: (i) Maasmechelen; (ii) northern and southwestern Germany; (iii) western Germany and Eupen; and (iv) Natoye (Figs. 1, 2). While for *nad*4, the *F*_*ST*_ values between Eupen and western Germany were not significantly different from zero, those for the microsatellites were almost all significant (Table 4).

**Table 4:** Population pairwise *F*_*ST*_ estimates per population, calculated using Arlequin v3.5

Collection locations	(1)	(2)	(3)	(4)	(5)	(6)	(7)	(8)	(9)
(1) Western Germany	0	0.065*	0.125*	0.120*	0.130*	0.174	0.222*	0.182*	0.164*
(2) Southwestern Germany	0.072	0	0.094*	0.152*	0.142*	0.171	0.260*	0.157*	0.226*
(3) Northern Germany	0.318*	0.199*	0	0.283*	0.174*	0.263	0.327*	0.164*	0.158*
(4) Eupen 2017	0.145	0.074	0.329	0	0.219*	0.368	0.488*	0.329*	0.354*
(5) Eupen 2018	0.542	0.367*	0.441*	0.759*	0	0.184	0.379*	0.280*	0.258*
(6) Eupen 2019	− 0.102	− 0.175	0.147	0.001	0.506	0	0.310	0.320	0.312*
(7) Maasmechelen	0.228	0.128	0.397*	0.001	0.856*	0.001	0	0.337*	0.311*
(8) Natoye 2012–2013	0.380*	0.259*	0.130*	0.342*	0.475*	0.204	0.380*	0	0.116*
(9) Natoye 2017–2019	0.547*	0.439*	0.221*	0.537*	0.651*	0.473	0.549*	0.128*	0

*Significant values at *P* < 0.0005 after standard Bonferroni correction: below diagonal, based on *nad*4; above diagonal, based on the microsatellites

**Fig. 3:**
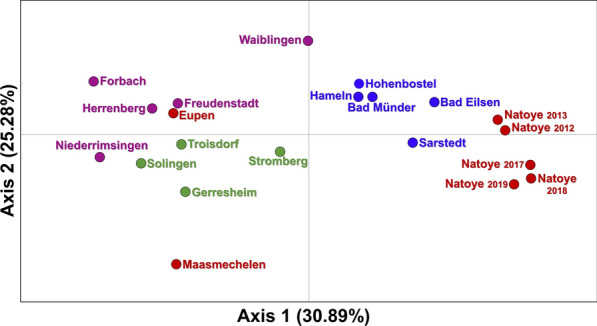
Principal coordinates analysis (PCoA) performed with GenAlEx v6.51b2 (first two axes explaining 56.18% of the genetic variability). Red indicates Belgian sampling localities; blue, violet and green indicates northern Germany, southwestern Germany and western Germany sampling localities, respectively

Three *nad*4 haplotypes were found in Eupen (H1, H5, H6), which also occurred in the western and southwestern German populations (Table 2). Eupen did not show a heterozygote excess (*P* > 0.05) using Bottleneck, nor did the German populations. Based on the microsatellite loci, Maasmechelen displayed significant pairwise *F*_*ST*_ values with all other populations (*F*_*ST*_ of 0.237; Table 4), except with the population at Eupen in 2019. On the PCoA (Fig. 3), Maasmechelen stands apart from all other populations; it also had the lowest allelic richness (2.143; Table 3) and only one *nad*4 haplotype (H1; Table 2).

### Temporal analysis at Natoye

**Fig. 4:**
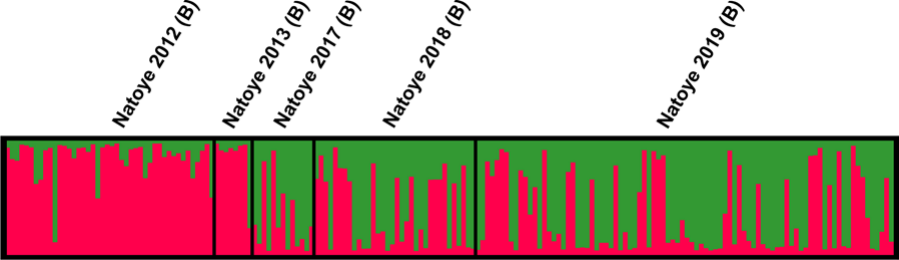
Clusters for *K* = 2 at Natoye (Belgium), inferred with Structure v2.3.4 software, after Evanno et al. [60] correction. The cluster membership of each individual is shown by the colour composition of the vertical lines, with the length of each coloured part of the line being proportional to the estimated membership coefficient

Bayesian cluster analysis based on the microsatellite data identified two admixing, genotypic clusters at Natoye (highest posterior probability for *K* = 2) (Fig. 4, Additional file 3: Fig. S3): the first one including the individuals collected in 2012–2013 and the second one including the individuals collected in 2017–2019. The first cluster has a predominant genotypic signal “red”, whereas the second cluster has a predominant genotypic signal “green” (Fig. 4). The *F*_*ST*_ values corroborate this structure, with no significant genetic differentiation between 2012 and 2013 or between 2017, 2018 and 2019, but with five of the six comparisons, 2012–2013* versus* 2017–2019, showing significant *F*_*ST*_ values (Table 5). The Bottleneck results indicated that the population of Natoye showed a significant heterozygosity excess in 2012–2013, but not in 2017–2019; however, *F*_*IS*_ estimates were significant in both cases (Table 3). In 2012–2013, the allelic richness was also lower, and there were fewer private alleles than in 2017–2019 (Table 3). Considering all *nad*4 data at Natoye, the most common haplotype was H9 over all years, detected 82 times, followed by H1 (*N* = 15). Three haplotypes were each only detected in 1 year at Natoye, namely H23 in 2012, H5 in 2017 and H47 in 2019 (Table 2).

**Table 5:** Population pairwise *F*_*ST*_ results between years at Natoye, calculated using Arlequin v3.5 based on microsatellite data

Year	2012	2013	2017	2018	2019
2012	0				
2013	− 0.020	0			
2017	0.111*	0.120*	0		
2018	0.101*	0.121	0.005	0	
2019	0.116*	0.132*	0.021	0.005	0

*Significant at *P* < 0.0005 after standard Bonferroni correction

## Discussion

The present results indicate that the Natoye population is significantly differentiated from all other populations considered in this study, both for *nad*4 and for the microsatellite data, with a high prevalence of *nad*4 haplotype H9 (80.4%, excluding individuals displaying potential mtDNA heteroplasmy). This haplotype also occurs in the USA, Germany, Austria, the Netherlands and Slovenia [15, 24, 42, 44], but has never been found in such high frequencies, except in the population of Pennsylvania (USA) in 1999–2000 where 62.6% of the individuals were recorded with H9 (*N*_TOT_ = 32) [44]. However, as the *nad*4 data did not show any geographical relationships, the source area(s) of the original introduction at Natoye remain elusive. The lack of structure also observed in previous studies is likely linked to the randomness of international introduction events [42–44], with specimens possibly originating from diverse populations.

The Natoye population also showed a clear difference between its genotypic microsatellite make-up in 2012–2013 and 2017–2019, i.e. before and after the elimination campaign which started in 2012 and ran till 2015 (no specimen was caught during routine surveillance in 2015–2016), as suggested by the significant *F*_*ST*_ values and the Bayesian clustering in Fig. 4. This was, however, not accompanied by a difference in the *nad*4 data (Table 2). In 2017–2019, the population had an increased allelic richness and number of private alleles compared to 2012–2013 (*P*_*a*_: 10 *versus* 1, respectively; Table 3). Between 2017 and 2019, 59 specimens displayed one or more private alleles, while only three specimens were recorded with a private allele in the time period 2012–2013. These latter results would indicate that there may have been one or multiple additional new introduction(s) from external source(s) at Natoye, which occurred after the elimination campaign. Multiple introductions seem to be common to pests associated with human-mediated transport [44, 67], which has an impact on the genetic composition of populations. While the present genetic study cannot provide further insights on the possible origin(s) of the new introduction event(s), the investigation of the trading history at the Natoye company indicates that tyres are regularly imported from an area in Germany colonised by *Ae. japonicus* only in 2017 (Elz, in the federal state of Hesse) ([39, 51]; personal comment H. Kampen and R. Müller). At the time of the investigation, samples from Hesse were not available for the present molecular work. Further investigation in this direction would possibly allow confirmation of this hypothesis.

The Natoye population present in 2012–2013 is, however, believed to have survived since a shift in the genetic signature before and after the elimination campaign was identified based on microsatellite data, but without complete replacement (Fig. 4; shift in the frequency of individuals from predominantly red in 2012–2013 to predominantly green in 2017–2019). The forest next to the premises of the tyre-trading company, where *Ae. japonicus* was collected during different monitoring projects, might have acted as a refuge [30]. Indeed, in its natural distribution range in East Asia, the species is usually found in forested areas [68], with breeding sites mainly distributed in urban and suburban area, while adults are more distributed in the forest [69]. Even if tree holes and other breeding sites had been neutralised during the elimination campaign (2012–2015) within the surrounding forests (by mechanical removal and larviciding of breeding sites, or filling tree holes with sand), a residual population could have survived at a low density, below the detection limit. The field monitoring results at Natoye also indicated a strong species abundance increase in 2019 (*N*_collected_ = 1725, whole season) compared to 2017 (*N*_collected_ = 31, collected over half a season) and 2018 (*N*_collected_ = 251, whole season), with evidence of a spread in the southwest direction in 2019 using the forest as a ‘shrub-corridor’ (Deblauwe et al., unpublished report). Several studies indicate that *Ae. japonicus* uses forest edges to spread [9, 70, 71]. This southwest spreading pattern was also observed in 2012 [17], but the current spread seems to be faster than in the past (Deblauwe et al., unpublished report). A new control campaign at Natoye was started in 2020 to control population density.

A few individuals collected in Natoye in 2008 and 2010 (*N*_TOT_ = 18) were previously analysed based on the same set of microsatellite loci in [42] and showed the lowest genetic diversity of all populations examined in the latter study, which included samples from Germany, Switzerland, Austria and Slovenia [42]. Although this low genetic diversity may be biased by the limited sampling, the genetic diversity estimates of Natoye in the present study covering the period 2012–2013 are in line with this previous finding when compared to expanding German *Ae. japonicus* populations (*A*_*R*_ = 3.286; Table 3). The individuals from Natoye (*N*_2012–2013_ = 52) were collected over the whole activity season of the mosquito, and also from a 2-km-wide perimeter around the tyre company site, which minimises the risk of biases due to relatedness.

Considering that both the sample sizes and the number of DNA markers used to investigate the genetic diversity of the Belgian populations along the border between Begium and Germany were limited, the results should be interpreted cautiously and should consider information collected in the field during the monitoring campaign. Additionally, the observed possible relatedness of the specimens cannot be dismissed without some reflection. For example, despite intensive monitoring efforts during the whole season, adult *Ae. japonicus* were only trapped twice at Maasmechelen, i.e. on 19 June and 3 July 2018 (using a Frommer updraft gravid trap (John W. Hock Co., Gainesville, FL, USA) (Deblauwe et al., unpublished report). Since these two trapping dates are close to each other, it is possible that the specimens derived from the same single introduction and eventually reproduced on site. The observed population genetic structure might therefore result from a strong genetic drift (Fig. 1). This assumption is supported by the presence of only one *nad*4 haplotype at Maasmechelen (H1). It is therefore not possible to make any further inferences about the potential origin of these specimens.

Despite the extensive sampling efforts in the allotment garden at Eupen (Deblauwe et al., unpublished report), only a few *Ae. japonicus* specimens were collected—once in 2017 (September), seven times in 2018 (June, July, August and September) and three times again in 2019 (May, June and July). The larvae collected in 2017 were most likely siblings as they were collected on the same date and at the same spot. In 2018, all life stages were collected in and around the allotment garden, while only larvae were found in 2019. Considering the monitoring efforts, these results indicate summer reproduction but the species is not believed to have established and overwintered yet, which rather points to multiple introductions at Eupen. Population clustering results based on microsatellite data at *K* = 6 and the PCoA suggest a relation between Eupen and the population of western Germany (Figs. 1–3), which is in agreement with the prediction that the species might cross the border with Belgium [39]. Whether this occurs* via* passive human-mediated ground transport or, alternatively, by natural spread cannot be determined as yet from the current dataset.

To further investigate the population genetic relationships, gain insight in the introduction pathways and investigate changes in the allelic frequencies over time in the frame of surveillance and elimination programmes, thorough sampling of all *Ae. japonicus* populations, including representatives of its native and invasive ranges, in additional to the use of genome wide genetic data, would be required.

## Conclusion

Considering the international movement of goods and people, the colonising behaviour of *Ae. japonicus* in Germany, its recent establishment in Luxembourg, the increasing population densities in Germany and Belgium [13, 14] and the relatedness of the population in Eupen with the one across the border in the western part of Germany, it is to be expected that further introductions will occur into Belgium. The eastern border of Belgium is at the front of the invasion of *Ae. japonicus* toward the west, while the present results also show that the elimination campaign undertaken over years at Natoye was not completely successful, which underlines the complexity of controlling invasive species. Sensibilisation along the German and Luxembourg border and control through larviciding and mechanical removal of breeding sites at the tyre-trading company in Natoye could help keeping densities and spread as low as possible.

## Supplementary Information

**Additional file 1: Fig. S1. **Results of the Bayesian clustering analysis with Structure v2.3.4 software, reporting the *ΔK* values calculated according to Evanno et al. [60] with the CLUMPAK web server.

**Additional file 2: Fig. S2. **Map of Belgium and Germany displaying the clustering analysis results for* K* = 2, based on our microsatellite database per sampling locality (each pie chart [dot] represents one location, colours of the pie chart represent the mean assignment probabilities for all individuals collected at that location to each clusters).

**Additional file 3: Fig. S3. **Results of the Bayesian clustering analysis with Structure v2.3.4 software at Natoye, reporting the *ΔK* values calculated according to Evanno et al. [60] with the CLUMPAK web server.

## Data Availability

The datasets supporting the conclusions of this article are included within the article.
