# Supplementary figures and images for: Population genetic structure of the Asian bush mosquito, Aedes japonicus (Diptera, Culicidae), in Belgium suggests multiple introductions
Source: Parasit Vectors. 2021 Mar 25;14:179. doi: 10.1186/s13071-021-04676-8 (PMC7995749; doi:10.1186/s13071-021-04676-8)

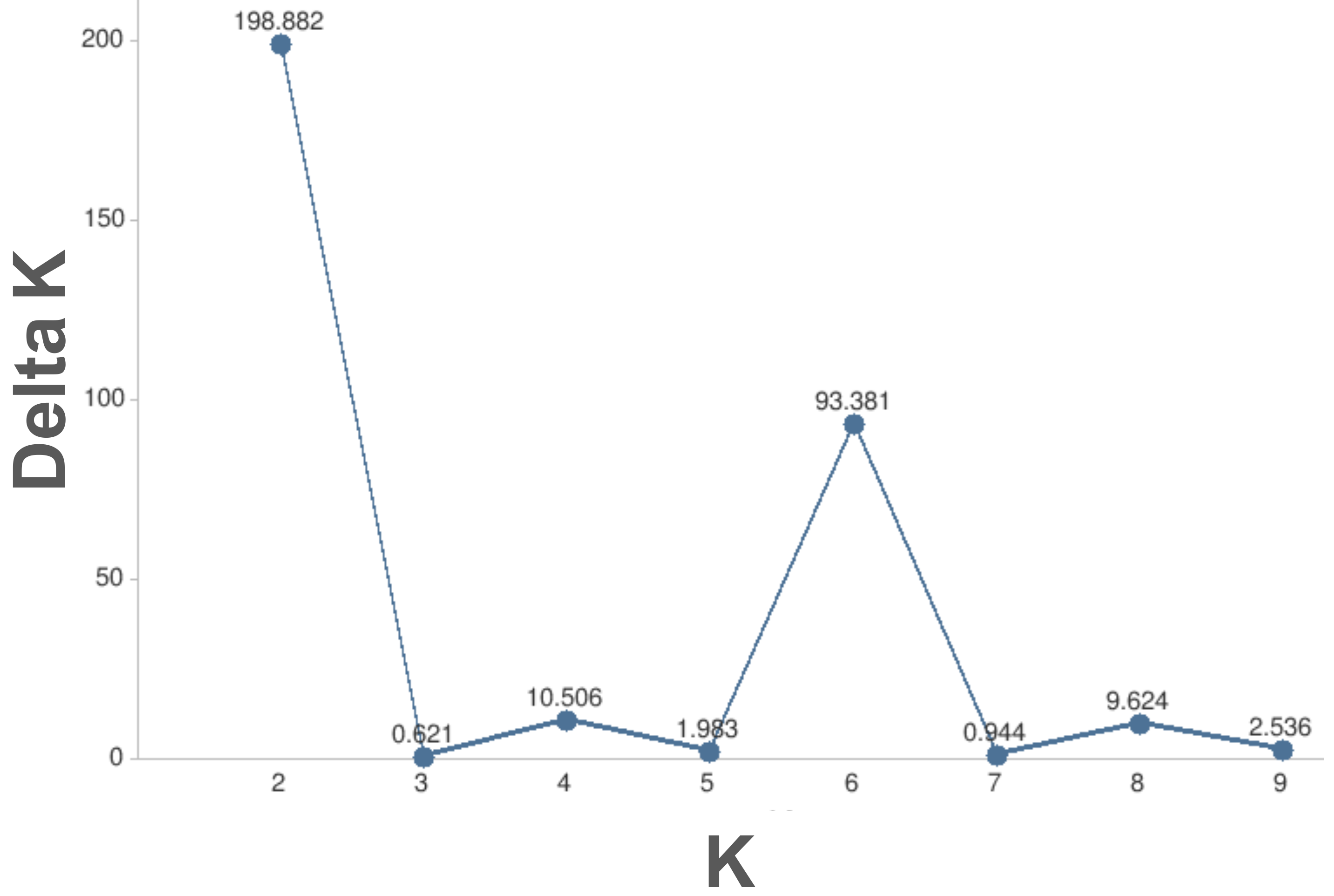

Supplement: Supplementary file 1 — Additional file 1: Fig. S1. Results of the Bayesian clustering analysis with Structure v2.3.4 software, reporting the ΔK values calculated according to Evanno et al. [60] with the CLUMPAK web server. [file 13071_2021_4676_MOESM1_ESM.png]

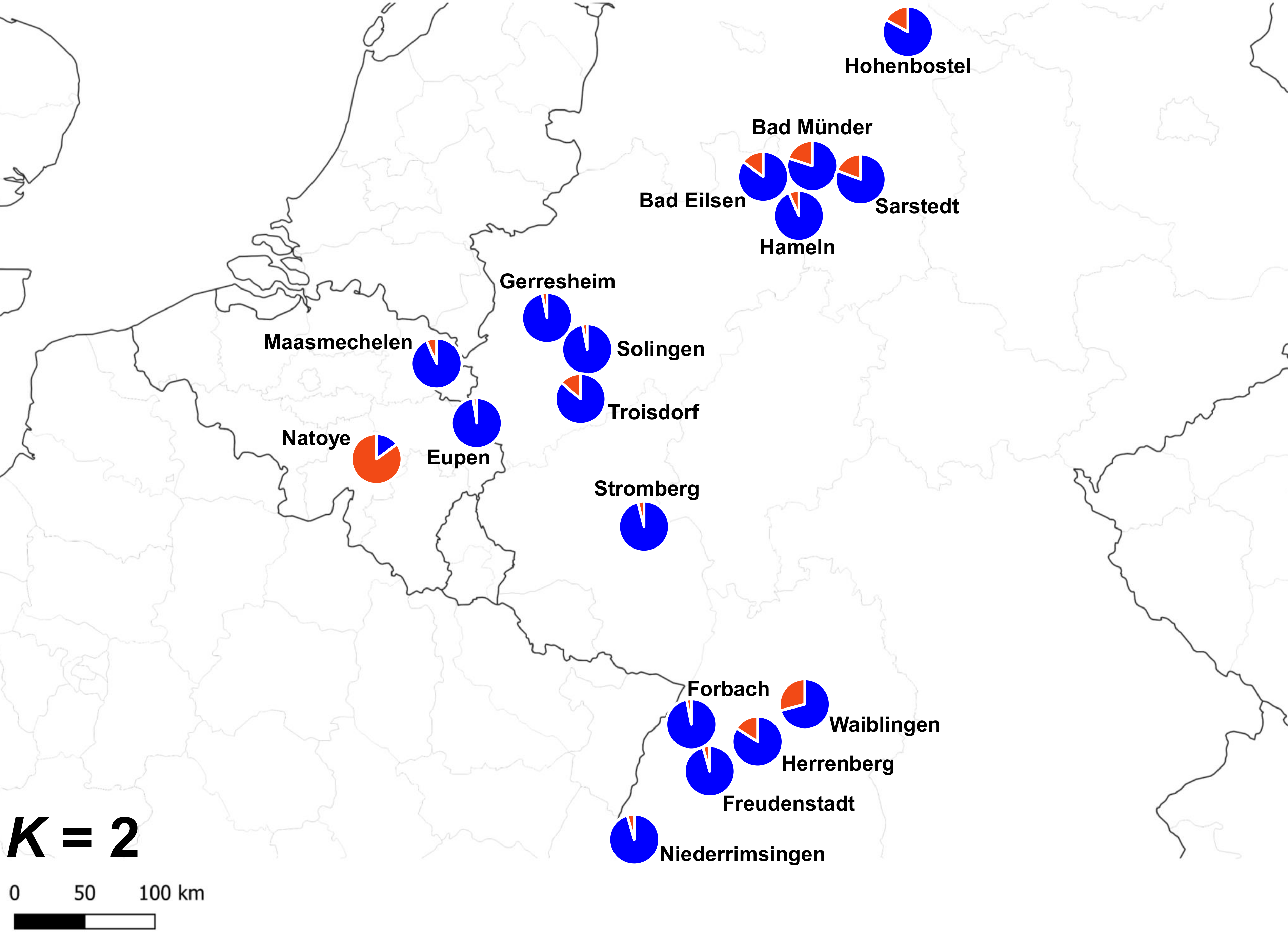

Supplement: Supplementary file 2 — Additional file 2: Fig. S2. Map of Belgium and Germany displaying the clustering analysis results for K = 2, based on our microsatellite database per sampling locality (each pie chart [dot] represents one location, colours of the pie chart represent the mean assignment probabilities for all individuals collected at that location to each clusters). [file 13071_2021_4676_MOESM2_ESM.png]

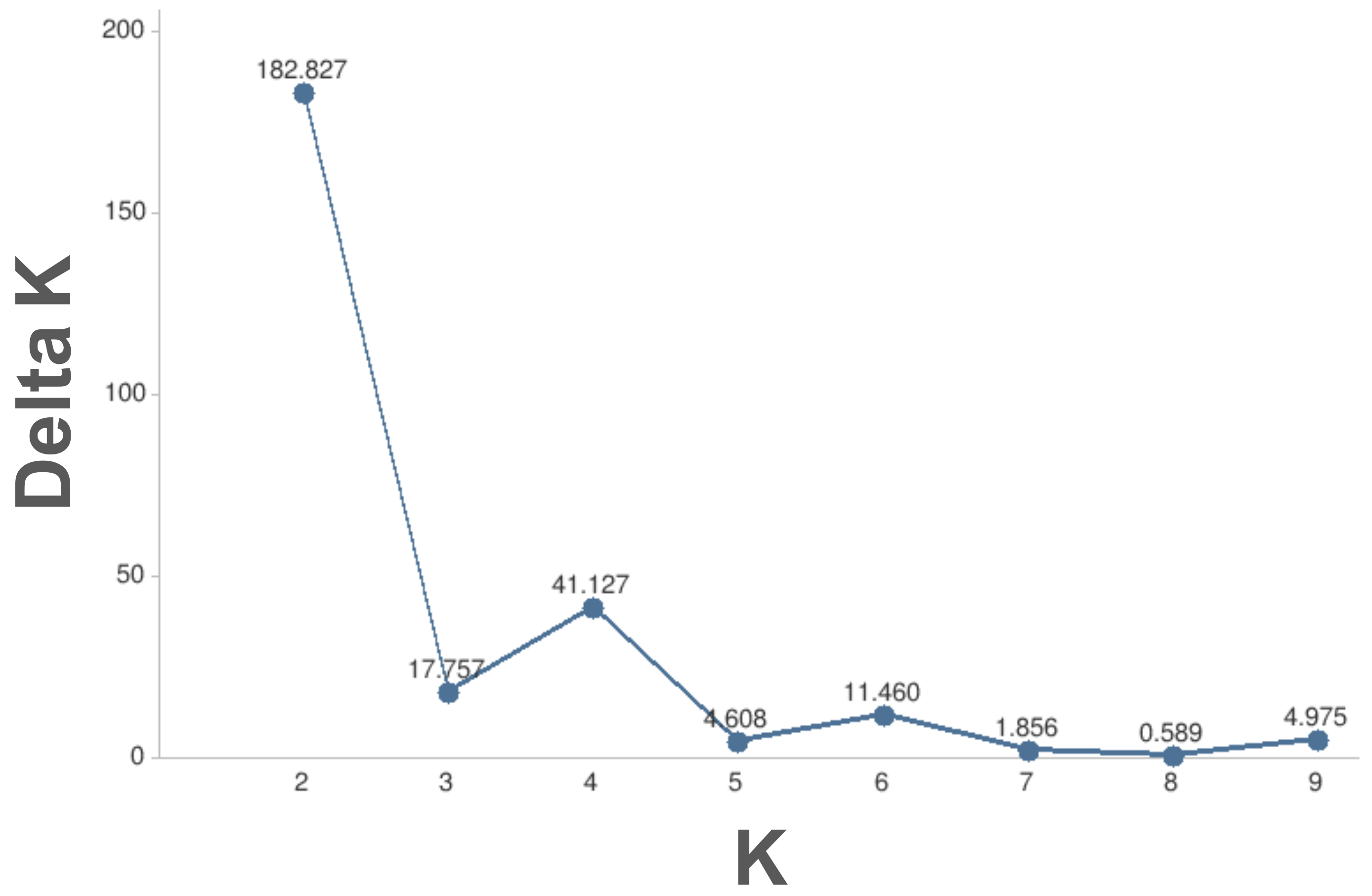

Supplement: Supplementary file 3 — Additional file 3: Fig. S3. Results of the Bayesian clustering analysis with Structure v2.3.4 software at Natoye, reporting the ΔK values calculated according to Evanno et al. [60] with the CLUMPAK web server. [file 13071_2021_4676_MOESM3_ESM.png]
